# NFIL3 and its immunoregulatory role in rheumatoid arthritis patients

**DOI:** 10.3389/fimmu.2022.950144

**Published:** 2022-11-11

**Authors:** Juping Du, Liyuan Zheng, Shuaishuai Chen, Na Wang, Xia Pu, Die Yu, Haixi Yan, Jiaxi Chen, Donglian Wang, Bo Shen, Jun Li, Shaobiao Pan

**Affiliations:** ^1^ Department of Clinical Laboratory, Taizhou Hospital of Zhejiang Province affiliated to Wenzhou Medical University, Linhai, China; ^2^ Department of Rheumatology and Immunology, Taizhou Hospital of Zhejiang Province Affiliated to Wenzhou Medical University, Linhai, China

**Keywords:** rheumatoid arthritis (RA), NFIL3, immune regulation, GEO, bioinformatics analysis

## Abstract

Nuclear-factor, interleukin 3 regulated (NFIL3) is an immune regulator that plays an essential role in autoimmune diseases. However, the relationship between rheumatoid arthritis (RA) and NFIL3 remains largely unknown. In this study, we examined NFIL3 expression in RA patients and its potential molecular mechanisms in RA. Increased NFIL3 expression levels were identified in peripheral blood mononuclear cells (PBMCs) from 62 initially diagnosed RA patients and 75 healthy controls (HCs) by quantitative real-time PCR (qRT-PCR). No correlation between NFIL3 and disease activity was observed. In addition, NFIL3 expression was significantly upregulated in RA synovial tissues analyzed in the Gene Expression Omnibus (GEO) dataset (GSE89408). Then, we classified synovial tissues into NFIL3-high (≥75%) and NFIL3-low (≤25%) groups according to NFIL3 expression levels. Four hundred five differentially expressed genes (DEGs) between the NFIL3-high and NFIL3-low groups were screened out using the “limma” R package. Enrichment analysis showed that most of the enriched genes were primarily involved in the TNF signaling pathway *via* NFκB, IL-17 signaling pathway, and rheumatoid arthritis pathways. Then, 10 genes (IL6, IL1β, CXCL8, CCL2, PTGS2, MMP3, MMP1, FOS, SPP1, and ADIPOQ) were identified as hub genes, and most of them play a key role in RA. Positive correlations between the hub genes and NFIL3 were revealed by qRT-PCR in RA PBMCs. An NFIL3-related protein–protein interaction (PPI) network was constructed using the STRING database, and four clusters (mainly participating in the inflammatory response, lipid metabolism process, extracellular matrix organization, and circadian rhythm) were constructed with MCODE in Cytoscape. Furthermore, 29 DEGs overlapped with RA-related genes from the RADB database and were mainly enriched in IL-17 signaling pathways. Thus, our study revealed the elevated expression of NFIL3 in both RA peripheral blood and synovial tissues, and the high expression of NFIL3 correlated with the abnormal inflammatory cytokines and inflammatory responses, which potentially contributed to RA progression.

## Introduction

Rheumatoid arthritis (RA) is a complex autoimmune and inflammatory disease characterized by specific autoantibodies, persistent synovitis, and symmetrical pain, swelling, and stiffness of the joints. In addition to joints, other tissues and organs, including blood vessels, lungs, mouth, and eyes, can also be affected due to persistent inflammation. Although the etiology of RA remains largely unknown, immune imbalances due to aberrant immune cells and innate and adaptive immune molecules have been considered key players in the pathogenesis of RA.

The transcription factor nuclear factor, interleukin 3 regulated (NFIL3) has been identified as a key immune regulator and is mainly expressed in natural killer (NK) cells, B lymphocytes, T lymphocytes, monocytes, and other immune cells (1, 2). Studies on the NFIL3^−/−^ mice revealed its essential role in the development and function of NK cells, CD8α^+^ dendritic cells, Th17 cells, and other innate lymphoid cells (3–7). Inhibition of NFIL3 expression in CD4^+^ T cells showed decreased IL-10 levels, damaged effector function, and more severe experimental autoimmune encephalomyelitis (8). IL-27-induced Tim3^+^ exhausted T cells exhibited a dysfunctional T-cell phenotype in an NFIL3-dependent manner (9). NFIL3 also suppressed the IL-12 p40 production in macrophages and was decreased in ulcerative colitis patients (10). The anti-inflammatory role played by NFIL3 in innate and adaptive immunity is starting to attract attention in the autoimmune field. NFIL3 was highly expressed in CD4^+^ T cells from systemic lupus erythematosus (SLE) patients compared with those from healthy controls, and NFIL3 inhibited the self-reactivity of T cells by decreasing CD40L expression (11). In addition, increased NFIL3 expression but reduced phosphorylation levels were observed in Tfh cells from SLE patients, resulting in excessive Tfh cell differentiation (12). A link between NFIL3 deficiency and juvenile idiopathic arthritis (JIA) has been identified in monozygotic twins who both harbored homozygous mutations in NFIL3. The parallel mouse model confirmed that NFIL3-deficient mice displayed more severe inflamed joints and arthritis (13). Inconsistent results have been reported about the NFIL3 expression in RA CD4^+^ T cells by Lu’s group (11, 12), but the sample was relatively small (15 and 10). Thus, the relationship between NFIL3 and RA needs to be further explored.

In the present study, we investigated the NFIL3 expression in peripheral blood mononuclear cells (PBMCs) from newly diagnosed RA patients. Then, we used the Gene Expression Omnibus (GEO) database to demonstrate the NFIL3 expression in RA synovial tissues and investigate its coexpressed genes and related pathways.

## Materials and methods

### Subjects

**Figure 1:**
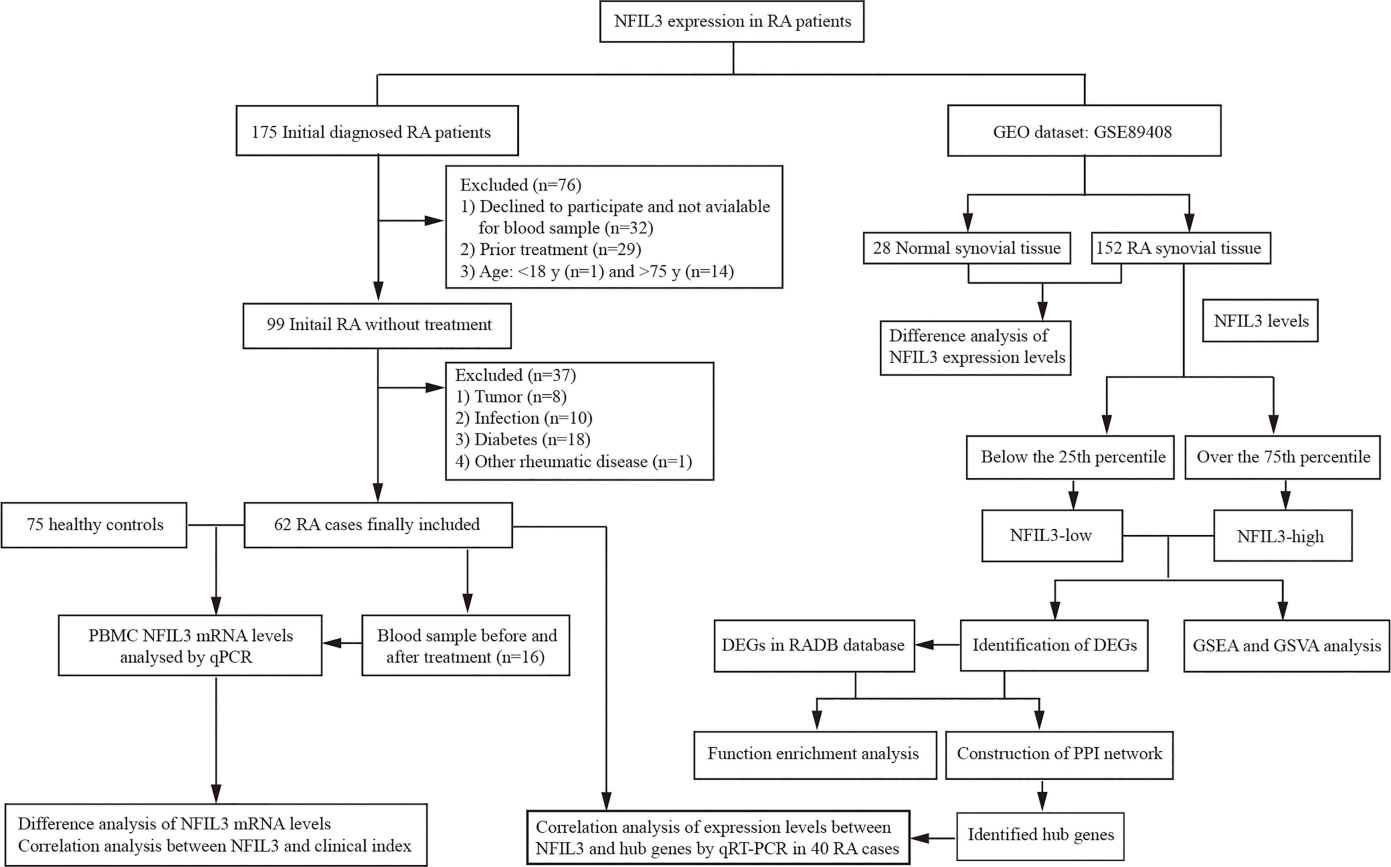
Overall workflow for analyzing NFIL3 expression in rheumatoid arthritis.

A total of 175 new RA patients who met the 2010 American College of Rheumatology/European Alliance of Associations for Rheumatology (ACR/EULAR) classification criteria of RA (14) were consecutively recruited at Taizhou Hospital of Zhejiang Province from December 2020 to October 2021. Patients who refused to participate and had prior treatment and a history of other rheumatic diseases, infection, diabetes, cancers, and inflammatory diseases were excluded from this study. According to the inclusion and exclusion criteria, finally, there were 62 initially diagnosed RA patients who were available for further analysis (**Figure 1** and **Table S1**). Clinical disease activity was scored according to the Disease Activity Score in 28 Joints System with Erythrocyte Sedimentation Rate (DAS28-ESR) and divided into three groups: the high activity group (DAS28-ESR > 5.1), moderate activity group (3.2 < DAS28-ESR ≤ 5.1), and low disease activity group (DAS28-ESR ≤ 3.2). Seventy-five healthy controls (HCs) were enrolled at the medical examination center. The study was approved by the Institutional Review Board of the Ethics Committee of the Taizhou Hospital of Zhejiang Province.

### Real-time quantitative PCR analysis

PBMCs were obtained from peripheral blood by Ficoll density gradient centrifugation (Sigma−Aldrich, Darmstadt, Germany). Total RNA was extracted from PBMCs using TRIzol reagent (Invitrogen, Carlsbad, CA, USA), and reverse transcription was performed using BeyoRT™ II First-Strand cDNA Synthesis Kit (Beyotime, Shanghai, China). NFIL3 and eight hub gene mRNA expression were measured *via* qRT-PCR with BeyoFast™ SYBR Green qPCR Mix (Beyotime, China). The amplification was conducted by a StepOnePlus™ System (Applied Biosystems, Foster City, CA, USA) following three-step PCR conditions: 95°C for 2 min, followed by 40 cycles of 95°C for 15 s and 60°C for 30 s, ending with 95°C for 15 s, 60°C for 15 s, and 95°C for 15 s. GAPDH was used as an endogenous control. The primers used in this study were listed in **Table S2** and synthesized by Sangon Biotech (Shanghai, China).

### Analysis of NFIL3 expression in rheumatoid arthritis synovial tissues in the gene expression omnibus database

Due to difficulties in obtaining synovial tissues, we collected gene expression data for RA synovial tissues from the GEO database to analyze NFIL3 expression. The sample size of most RA synovial tissue datasets in GEO was small except for GSE89408, which contains 152 RA synovial tissues and 28 normal control tissues. Thus, we chose the GSE89408 dataset for further analysis. Patients with less than or equal to 25% expression levels of NFIL3 were defined as NFIL3-low, while patients with more than or equal to 75% expression levels of NFIL3 were defined as NFIL3-high. Differential expression analysis between the RA and HC groups and between the NFIL3-high and NFIL3-low groups was conducted using the *limma* package of R language (version 4.1.2). Differentially expressed genes (DEGs) with an adjusted p-value <0.05 and |log_2_ fold change| (|log_2_ FC|) > 1 were considered significant.

Then, Gene Ontology (GO) and Kyoto Encyclopedia of Genes and Genomes (KEGG) enrichment were analyzed with the DEGs based on the DAVID database (https://david.ncifcrf.gov/ ) and Pathview database (https://pathview.uncc.edu/ ). Gene set variation analysis (GSVA) using the MSigDB hallmark gene set file “c2.cp.kegg.v7.5.symbols” and gene set enrichment analysis (GSEA) was also performed to further explore the NFIL3-related signaling pathways. A protein–protein interaction (PPI) network of genes that coexpressed with NFIL3 was constructed with the Search Tool for the Retrieval of Interacting Genes (STRING: https://cn.string-db.org/ ) and visualized by Cytoscape software (version 3.8.1). The top 10 hub genes and main gene modules in the PPI network were screened out by MCODE and cytoHubba in Cytoscape. qRT-PCR of the hub genes was performed in PBMCs of some newly diagnosed RA patients mentioned above (**Table S1**) to confirm the linkage between NFIL3 and the hub genes.

We also intersected RA-related genes in the RADB database (15) (http://www.bioapp.org/RADB/, a database of rheumatoid arthritis-related polymorphisms) with NFIL3-related DEGs. KEGG pathway enrichment and the PPI network were analyzed for the RA- and NFIL3-related DEGs.

### Statistical analysis

The data are presented as the median ± SD. Relative NFIL3 mRNA expression in the RA and HC groups was analyzed with Student’s t-test by SPSS version 26.0 (SPSS Inc., Chicago, IL, USA). Paired Student’s t-test was used to measure the significance of expression change before and after treatment. The graphs were plotted with GraphPad Prism 8.0 (GraphPad Prism Software Inc., San Diego, CA, USA) and R software (version 4.1.2). Significance was set as p < 0.05.

## Results

### NFIL3 expression levels in rheumatoid arthritis patients and healthy controls

No significant difference in gender distribution was observed between the RA and HC groups (**Table 1** and **Table S1**). The median age was significantly higher in the RA group than in the HC group (53.3.5 ± 10.0 *vs.* 49.6 ± 10.6, p < 0.05). The disease duration in newly diagnosed RA patients was relatively short, and the proportion of patients with a disease course of fewer than 6 months was as high as 72.6% (45/62). Up to 80.6% (50/62) of patients were defined as having moderate and high disease activities.

**Table 1:** Clinical characteristics of RA patients and controls.

Variables	Healthy control (n = 73)	Rheumatoid arthritis (n = 62)			
		All (n = 62)	DAS28-ESR ≤ 3.2 (n = 13)	3.2 < DAS28-ESR ≤ 5.1 (n = 25)	DAS28-ESR > 5.1 (n = 24)
Female, n (%)	48 (65.8)	43 (69.4)	8 (61.5)	20 (80.0)	15 (62.5)
Age (years)	49.6 ± 10.6	53.3 ± 10.0	46.3 ± 10.2	54.2 ± 7.7	56.3 ± 10.4
Duration (m)	–	3.0 (2.0–12.0)	4.0 (3.0–10.0)	3.0 (2.0–12.0)	3.0 (2.0–10.5)
RF (KU/L)	–	72.2 (18.5–175.1)	52.2 (9.0–212.9)	72.4 (25.4–158.4)	72.7 (14.1–214.9)
Anti-CCP (U/ml)^*^	–	89.6 (12.2–200.0)	200.0 (0.5–200.0)	70.4 (18.2–200.0)	84.5 (5.4–200.0)
ESR (mm/h)	–	37.5 (21.5–54.0)	17.0 (4.5–28.0)	36.0 (19.5–46.5)	53.5 (33.5–68.8)
DAS28-ESR	–	4.50 (3.33–5.58)	2.59 (2.29–2.90)	4.25 (3.76–4.67)	5.94 (5.51–6.38)
Stiffness, n (%)	–	27 (43.5)	1 (7.7)	11 (44.0)	15 (62.5)

*Six RA patients lack anti-CCP antibody data. TJC28, Tender Joint Count 28 Joints; SJC, Swollen Joint Count 28 Joints; RF, rheumatoid factor; anti-CCP, anti-cyclic citrullinated peptide; ESR, erythrocyte sedimentation rate. DAS28-ESR, Disease Activity Score in 28 Joints System with Erythrocyte Sedimentation Rate, formula: DAS28-ESR = [0.56 * sqrt(TJC28) + 0.28 * sqrt(SJC28) + 0.70 * Ln(ESR)] * 1.08 + 0.16.

**Figure 2:**
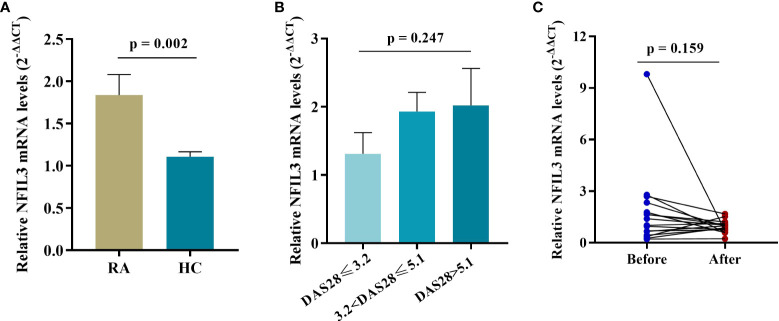
NFIL3 expression in PBMCs from newly diagnosed RA patients and healthy controls. **(A)** Comparison of NFIL3 expression between RA and HC groups using qRT-PCR. **(B)** Differences in NFIL3 expression among RA patients with low, moderate, and high disease activity. **(C)** Alterations of NFIL3 expression before and after treatment. RA patients were divided into three groups according to DAS28-ESR score. Low disease activity, DAS28 ≤ 3.2; moderate disease activity, 3.2 < disease activity ≤ 5.1; high disease activity, DAS28 > 5.1. GAPDH was used as a reference gene for data normalization. PBMCs, peripheral blood mononuclear cells; RA, rheumatoid arthritis; HC, healthy control.

NFIL3 mRNA expression was significantly higher in RA PBMCs than in HC PBMCs (**Figure 2**). An increasing trend in NFIL3 expression was detected along with the severity of disease activity, but there were no significant differences. In addition, no significant change in NFIL3 mRNA levels was detected before or after treatment (**Table S3** and **Figure 2C**).

### Correlation between NFIL3 levels and rheumatoid arthritis clinical data

**Figure 3:**
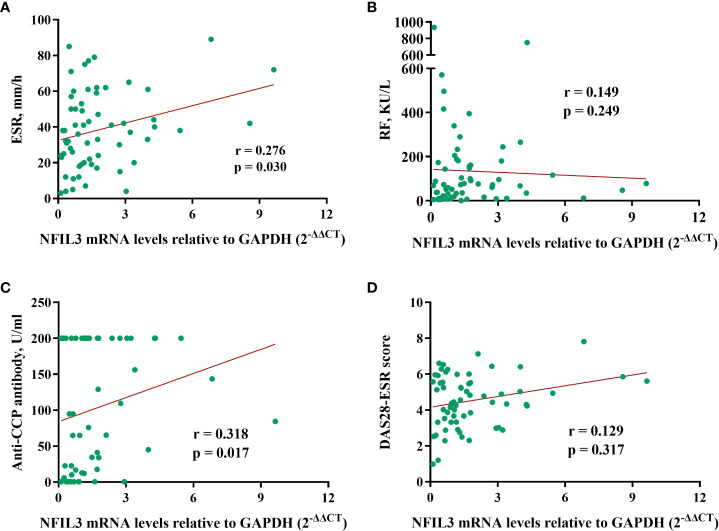
Correlations between NFIL3 expression and clinical indexes. **(A)** ESR, **(B)** RF, **(C)** anti-CCP, and **(D)** DAS28-ESR. ESR, erythrocyte sedimentation rate; RF, rheumatoid factor; anti-CCP, anti-cyclic citrullinated peptide; DAS28-ESR, disease activity score with 28 joints using erythrocyte sedimentation rate.

The correlation between NFIL3 and clinical laboratory data was investigated (**Figure 3**). We found a weak correlation between NFIL3 and anti-cyclic citrullinated peptide (anti-CCP) antibodies (r = 0.318, p = 0.0117) and the erythrocyte sedimentation rate (ESR) (r = 0.276, p = 0.030). However, no relationship between NFIL3 and RF or DAS28-ESR was observed (all p > 0.05). Only 32 patients had lipid metabolism-related indices, and we found no correlation between NFIL3 expression and total glyceride, total cholesterol, apolipoprotein (a)/(b), low-density lipoprotein cholesterol, or low-density lipoprotein cholesterol (data not shown).

### Identification of differentially expressed genes in different NFIL3 subgroups

We identified 9,379 DEGs in RA synovial tissue based on DESeq analysis from GSE89408 datasets compared with the normal controls (data not shown), which revealed that NFIL3 was significantly upregulated in RA patients (logFC = 1.18, adj.p = 1.61 × 10^−27^, **Figure S1**). To further understand the molecular biology of NFIL3 in RA, we analyzed the DEGs between the high- and low-NFIL3 expression groups. A total of 405 genes (upregulated, 191; downregulated, 214) showed significant differential expression between these two groups (**Table S4**).

### Function enrichment analysis and the protein–protein interaction network

**Figure 4:**
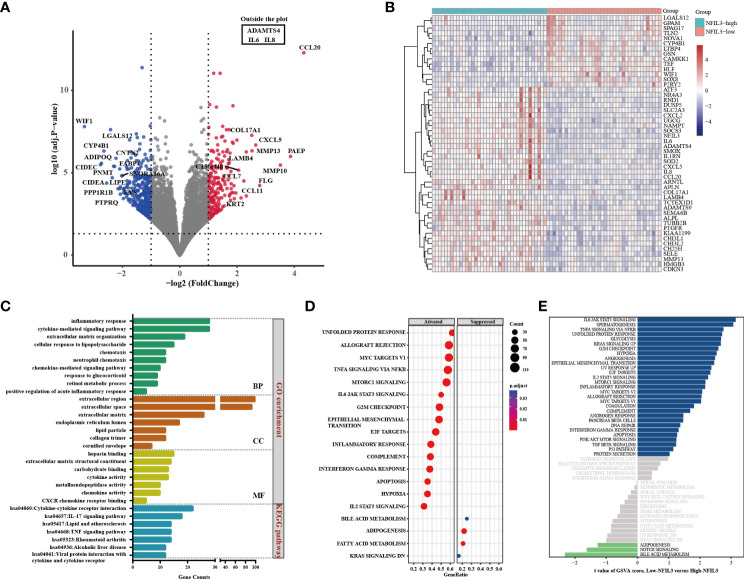
Identification of DEGs between the NFIL3-high and NFIL3-low groups and functional enrichment analysis. **(A)** Volcano plot shows the DEGs by comparing NFIL3-high and NFIL3-low synovial tissues from the GSE89408 dataset; red dot represents upregulated DEGs; blue dot represents downregulated DEGs; gray dot represents no significantly DEGs. **(B)** A heatmap of the 50 most significant DEGs according to the adjusted pvalue; blue indicates downregulated genes; red indicates upregulated genes. **(C)** GO enrichment and KEGG pathway analyses of the DEGs from DAVID database; the long bar represents the gene counts enriched in each pathway. **(D)** GSEA enrichment analysis shows that there are 15 upregulated and 4 downregulated gene sets with statistical significance between the high- and low-NFIL3 expression groups. **(E)** GSVA enrichment analysis of the 50 hallmark gene sets between the NFIL3-high and NFIL3-low groups. DEGs, differentially expressed genes; GSEA, gene set enrichment analysis; GSVA, gene set variation analysis. GO, Gene Ontology; KEGG, Kyoto Encyclopedia of Genes and Genomes.

GO and KEGG analyses of 425 genes that were coexpressed with NFIL3 were conducted using the DAVID database and revealed that the biological processes were primarily enriched in the inflammatory response, cytokine-mediated signaling pathway, and cellular response to lipopolysaccharide. For the molecular functions, NFIL3 was mainly enriched in chemokine activity, CXCR chemokine receptor binding, and metalloendopeptidase activity. KEGG pathway analysis showed that NFIL3-related genes were involved in the IL-17 signaling pathway, rheumatoid arthritis pathway, and TNF signaling pathway (**Figure 4**). We visualized the hsa05323 rheumatoid arthritis pathway using the online database Pathview and observed that the various proinflammatory cytokines, chemokines, and matrix metalloproteinases were highly expressed in the NFIL3-high group (**Figure S2**). Moreover, KEGG enrichment analysis showed that the 191 upregulated DEGs were mainly associated with inflammation-related pathways and that the 214 downregulated DEGs were significantly associated with metabolic pathways and PPAR signaling pathways (**Figure S3**). To further examine the relationship between NFIL3 expression and the enriched pathways, GSEA and GSVA were performed and showed that IL-6 JAK STAT3 signaling, spermatogenesis, and TNFα signaling *via* NFκB and glycolysis pathways were significantly enriched in the NFIL3-high group (**Figure 4**).

**Figure 5:**
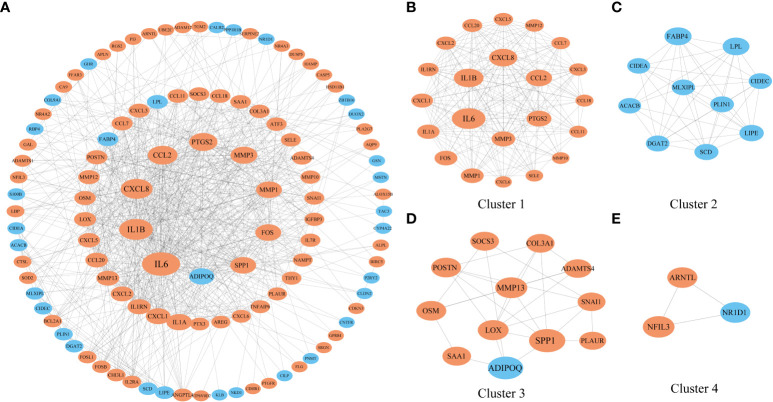
Construction of a PPI network of genes that coexpressed with NFIL3. **(A)** The PPI network with 112 nodes and 719 edges was constructed based on the STRING dataset and visualized by Cytoscape. **(B–E)** Four significant clusters were identified from the network by MCODE. The nodes with orange color represent the upregulated genes, while those with blue color represent the downregulated genes. The shape size of the nodes is arranged by the degree in the network. PPI, protein–protein interaction.

Then, we extracted genes that were coexpressed with NFIL3 to construct the PPI network with the STRING database and identified the genes and the submodules most relevant to NFIL3 by MCODE and cytoHubba in Cytoscape software. Nine of the top 10 degrees of hub genes were increased expression in the NFIL3-high group, including interleukin 6 (IL-6), interleukin 1 beta (IL1B), C-X-C motif chemokine ligand 8 (CXCL8), C-C motif chemokine ligand 2 (CCL2), prostaglandin-endoperoxide synthase 2 (PTGS2), matrix metallopeptidase 3 (MMP3), matrix metallopeptidase 1 (MMP1), Fos proto-oncogene (FOS), and secreted phosphoprotein 1 (SPP1), while adipose most abundant gene transcript 1 protein (ADIPOQ) was significantly decreased. The major module and clusters of the PPI network are visualized in **Figure 5**. Four clusters identified by MCODE were significantly associated with the inflammatory response, lipid metabolic process, extracellular matrix organization, and circadian rhythm-related biological process. All genes except ADIPOQ in Cluster 1 and Cluster 3 were upregulated in the NFIL3-high group and involved in IL-17 signaling pathways and the type II diabetes mellitus pathway. The genes in Cluster 2 were downregulated and mainly involved in the PPAR signaling pathway.

### Genes coexpressed with NFIL3 in rheumatoid arthritis

To further explore the relationship between NFIL3-related genes and RA, we obtained 636 RA-related genes from RADB. There were 29 common genes (including IL1B, IL-6, CXCL8, CCL2, IL1A, MMP3, MMP1, IL1RN, PTGS2, and SPP1) between the NFIL3 coexpression genes and RA-related genes (**Figure S4**). We also constructed a network of the 29 common genes associated with both NFIL3 expression and RA (**Figure S4**). These genes were mainly enriched in the IL-17 signaling pathway, TNF signaling pathway, and rheumatoid arthritis pathways.

### Validation of the correlation between NFIL3 and hub genes

**Figure 6:**
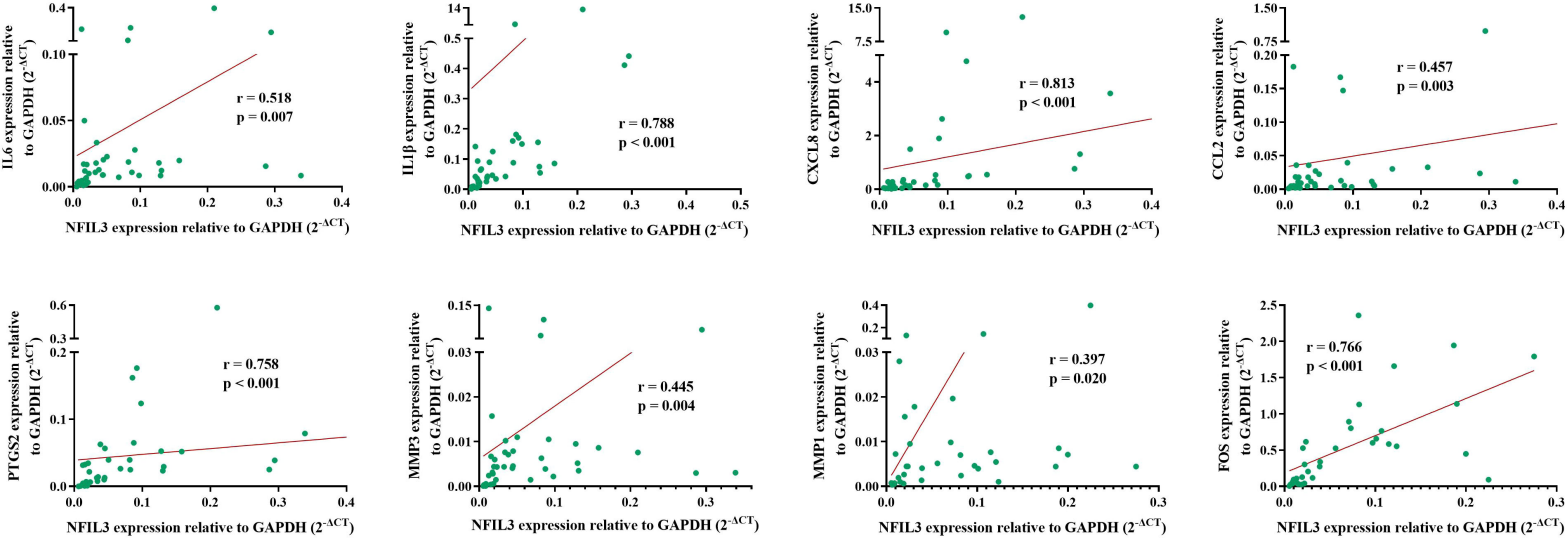
Association between NFIL3 and its coexpressed hub genes in RA. The eight hub genes and NFIL3 expression levels were analyzed in RA PBMCs by qRT-PCR and normalized to GAPDH. Spearman’s correlation analysis of NFIL3 and IL-6, IL1B, CXCL8, CCL2, PTGS2, MMP3, MMP1, and FOS in RA patients. RA, rheumatoid arthritis; PBMCs, peripheral blood mononuclear cells.

To verify the relationship between NFIL3 and hub genes, we analyzed eight hub genes (IL-6, IL1β, CXCL8, CCL2, PTGS2, MMP3, MMP1, and FOS) and NFIL3 expression in PBMCs from 40 out of 62 newly diagnosed RA patients using qRT-PCR. The two hub genes (MMP1 and FOS) were analyzed in 34 RA patients due to not enough cDNAs in six RA patients. qRT-PCR showed that expression levels of NFIL3 were positively correlated with all the eight hub genes, especially strong correlation observed with IL1β (r = 0.788, p < 0.001), CXCL8 (r = 0.813, p < 0.001), PTGS2 (r = 0.758, p < 0.001), and FOS (r = 0.766, p < 0.001) (**Figure 6**).

## Discussion

In the present study, increased mRNA NFIL3 levels were found in RA peripheral blood, and a correlation between NFIL3 and anti-CCP antibodies was also demonstrated. Moreover, we confirmed high NFIL3 expression in RA synovial tissue using the GEO dataset and identified NFIL3 coexpression genes, the potential pathways, and molecular functions, which were mainly involved in the IL-17 signaling pathway, lipid metabolism, and rheumatoid arthritis pathways. Finally, a significantly positive relationship between NFIL3 and proinflammatory molecules identified in the GEO dataset was established in RA PBMCs.

Previously, Lu’s group investigated NFIL3 expression in RA CD4^+^ T cells in two separate studies (11, 12). Compared to normal controls, no significant difference was observed in 15 RA patients using qRT-PCR (11), but the expression was increasingly detected in 10 RA CD4^+^ T cells using flow cytometry (12). In this study, we revealed elevated NFIL3 transcription levels in RA PBMCs compared to HC PBMCs. This inconsistency might be due to the different sample sizes and sample types. The immunosuppressive properties of NFIL3, including T-cell dysfunction, the inhibition of autoantibody production, and the induction of anti-inflammatory molecules, such as IL-10 and Tim-3, have been extensively established (8, 9, 11). Twins with NFIL3 deficiency developed JIA, and the protective role of NFIL3 in SLE patients has been reported (12, 13). These findings suggest an immune suppressive function of NFIL3 in autoimmune diseases. However, it remains largely unknown why NFIL3 expression is enhanced in RA and SLE patients. Based on previous research data, the following viewpoints might be raised to explain this phenomenon. First, NFIL3 might act as a negative feedback regulator to suppress inflammatory responses under autoimmune conditions. For instance, increased NFIL3 expression has been observed in Tfh cells but acts as a suppressor in Tfh cell differentiation. In addition, NFIL3 phosphorylation was essential for its immunoregulatory effect on NK cells (16) and T follicular helper (Tfh) cell development (12). Enhanced NFIL3 expression might compensate for damaged regulatory functions due to abnormal post-translational modifications. Reduced phosphorylation levels in SLE patients might support our hypothesis, which needs to be further verified. The high expression of NFIL3 has been well documented in T-cell activation and specific virus infection (9). Kim et al. reported that overexpression of NFIL3 in Treg cells impairs its suppressive activity by inhibiting foxp3 expression (17), which is crucial for RA pathogenesis.

Moreover, we evaluated changes in NFIL3 expression before and after treatment and found no significant differences. This finding was inconsistent with previous studies, which revealed that NFIL3 was essential for the glucocorticoid-induced anti-inflammatory response and apoptosis (11, 18–20). Seven (7/16, 43.8%) patients used glucocorticoids in our study and also showed no differences. This outcome might be due to our small sample size, most importantly the sample collection at various treatment times. The correlation between NFIL3 expression and RA treatment requires further analysis.

Synovial hyperplasia is one of the major clinical features of RA patients. High NFIL3 expression in RA synovial tissues was confirmed using a GEO dataset with large sample size, indicating that abnormal expression of NFIL3 may participate in RA development. Further functional enrichment analysis revealed that NFIL3 coexpression genes were strongly correlated with the inflammatory response, cytokine-mediated signaling pathway, cellular response to lipopolysaccharide, and response to glucocorticoid in biological processes and were significantly enriched in the IL-17 signaling pathway, TNF signaling pathway, and rheumatoid arthritis pathways referred to as KEGG pathway. IL-17 is a hallmark of Th17 cells and is highly expressed in RA patients (21, 22). It has been accepted that IL-17 enhances proinflammatory cytokine production and macrophage migration (23). The effect of NFIL3 on Th17 differentiation has been established in NFIL3^−/−^ mice with experimental autoimmune encephalitis (7, 8, 24). GSEA and GSVA also showed that the IL-6 JAK STAT3 signaling pathway, TNFA signaling *via* the NFκB pathway, IL-2 STAT5 signaling pathway, and MTORC1 signaling pathway were enriched in the NFIL3-high group. Most enrichment pathways have been considered to be involved in RA development. For instance, TNFα is an important proinflammatory cytokine that promotes the inflammatory cascade and plays a key role in RA pathogenesis (25–27). The clinical benefits of the TNF antagonist infliximab have been extensively reported in RA patients (26, 28). In addition, IL-6-mediated JAK/STAT3 signaling is essential for osteoclastogenesis, Th17 differentiation, and chronic inflammation and has a crucial effect on the development of RA (29–31). The JAK inhibitor tofacitinib and IL-6 inhibitor sarilumab have been used for the treatment of various autoimmune diseases, such as RA (32–34).

Interestingly, most downregulated DEGs in the NFIL3-high group participated in lipid metabolism and the PPAR signaling pathway. The interplay between immunity and dysregulated lipid metabolism is well documented (35, 36). PPARγ, the lipid regulator, exerts anti-inflammatory effects by inhibiting the activation of various immune cells, decreasing proinflammatory cytokines, and inducing macrophage polarization toward the M2 phenotype. Recent advances revealed that enhancing the expression of PPARγ could attenuate disease manifestations (37). The adipokine fatty acid binding protein 4 (FABP4), which is an increased expression in RA serum and synovial tissues (38–40), plays an important role in the regulation of inflammation. Guo et al. found that inhibition of FABP4 expression in macrophages could alleviate RA synovitis, angiogenesis, and cartilage degeneration (40). Studies of transcriptomes of epithelial cell-specific NFIL3 knockout mice showed declined FABP4 expression, suggesting a positive regulatory relationship (41).

Furthermore, a PPI network related to NFIL3 expression was constructed based on the STRING database, and 10 hub genes were found. Among the 10 hub genes, most were proinflammatory molecules that were increased in the NFIL3-high group. Consistent with these results, we confirmed the positive correlations between NFIL3 and proinflammatory molecule expression in RA PBMCs. Most hub genes have been reported to be essential inflammatory mediators in RA pathogenesis. For example, IL-6 and IL1B were confirmed to be involved in the regulation of the inflammatory response and the development of various autoimmune diseases, including RA, and were effective targets for treatment (42). Consistent with experiments on NFIL3-deficient murine bone marrow-derived macrophages and mice, the proinflammatory cytokines IL-6 and IL-1β were identified as downstream signaling molecules for NFIL3 (10, 13). Moreover, higher increases in chemokines and metalloproteinase (CXCL8, CCL2, SPP1, MMP1, and MMP3) were observed in the NFIL3-high group. These proinflammatory factors are mainly secreted by synovial fibroblasts and macrophages to promote inflammatory cell infiltration, synovial hyperplasia, and joint destruction. PTGS2 encodes cyclooxygenase 2 (COX2) and is the target of non-steroidal anti-inflammatory drugs (NSAIDs). The high expression of PTGS2 has been observed in RA synovial tissues (43). In addition, to participate in an inflammatory response, PTGS2 has a promotive effect on the proliferation of RA fibroblast-like synoviocytes (43). The inhibition role of NFIL3 on PTGS2 has been reported in MC3T3-E1 osteoblastic cells (44). In agreement with previous findings (2, 45), the major function of NFIL3 was associated with the inflammatory response, lipid metabolic process, extracellular matrix organization, and circadian rhythm, as identified by MCODE in Cytoscape. Furthermore, we found that 29 both RA- and NFIL3-related genes were correlated with the IL-17 signaling pathway and TNF signaling pathway, indicating that NFIL3 might be a major regulator of RA inflammation.

Several limitations in our study should be noted. First, the effect of treatment on NFIL3 expression should be further explored in a large sample and with a more rigorous experimental design. Second, we should obtain RA synovial tissues to confirm NFIL3 expression and posttranslational modification levels, such as phosphorylation levels, in the future. In addition, the exact downstream molecules of NFIL3 that contribute to RA development should be investigated.

In conclusion, our results showed that NFIL3 expression was significantly increased in RA PBMCs and synovial tissues. Potential key genes and pathways of NFIL3 in RA were identified using transcription expression data from the GEO dataset and verified in RA PBMCs. NFIL3 expression may correlate with the production of various proinflammatory cytokines (IL-6, IL1β, CXCL8, CCL2, PTGS2, MMP3, and MMP1), supporting its role in RA development.

## Data availability statement

The original contributions presented in the study are included in the article/supplementary material. Further inquiries can be directed to the corresponding authors.

## Ethics statement

The studies involving human participants were reviewed and approved by the Ethics Committee of the Taizhou Hospital of Zhejiang province. The patients/participants provided their written informed consent to participate in this study.

## Author contributions

JD, SP, and JL designed the study. SP provided the patient and clinical information. JD, LZ, SC, NW, XP, and DY performed the qRT-PCR and analyzed the data. HY, DW, JC, BS, and JL collected the samples and clinical information. JD wrote the first draft. All authors were involved in revising the manuscript and approved the submitted version.

## Funding

This study was supported by the Zhejiang Provincial Natural Science Foundation of China (LQ19H100001), Medical Science and Technology Project of Zhejiang Province (2017KY708, 2018KY913, and 2022KY1381), National Natural Science Foundation of China (81802078), and the Science and Technology Plan of Taizhou City (21ywb02).

## Acknowledgments

We thank Tianbin Tang for his assistance with bioinformatics analysis.

## Conflict of interest

The authors declare that the research was conducted in the absence of any commercial or financial relationships that could be construed as a potential conflict of interest.

## Publisher’s note

All claims expressed in this article are solely those of the authors and do not necessarily represent those of their affiliated organizations, or those of the publisher, the editors and the reviewers. Any product that may be evaluated in this article, or claim that may be made by its manufacturer, is not guaranteed or endorsed by the publisher.

## Supplementary material

Supplementary Figure 1 Up-regulation of NFIL3 expression in RA synovial tissues compared with normal tissues from the GSE89408 dataset.

Supplementary Figure 2 Rheumatoid arthritis pathway between NFIL3-high and NFIL3-low groups. The expression changes of DEGs related to the hsa05323 rheumatoid arthritis pathway were visualized by online database Pathview. Red indicated increased expression.

Supplementary Figure 3 KEGG analysis of the upregulated and downregulated DEGs. Up-regulated DEGs were mainly enriched in IL-17 signaling pathway and cytokine-cytokine receptor interaction pathways. Down-regulated DEGs were mainly enriched in metabolic pathways.

Supplementary Figure 4 The heatmap and PPI network of 29 RA- and NFIL3-related genes. There are 29 overlapping genes between the NFIL3 co-expression genes and the RA-related genes from the RADB database. **(A)** A heatmap was applied to visualized the expression differences of 29 RA-related genes in the NFIL3-high and NFIL3-low groups. Blue indicates down-regulation; red indicates up-regulation. **(B)** The PPI network contains 20 nodes and 117 edges. The expression of all the nodes were upregulated.
