# Supplementary figures and images for: NFIL3 and its immunoregulatory role in rheumatoid arthritis patients
Source: Front Immunol. 2022 Nov 11;13:950144. doi: 10.3389/fimmu.2022.950144 (PMC9692021; doi:10.3389/fimmu.2022.950144)

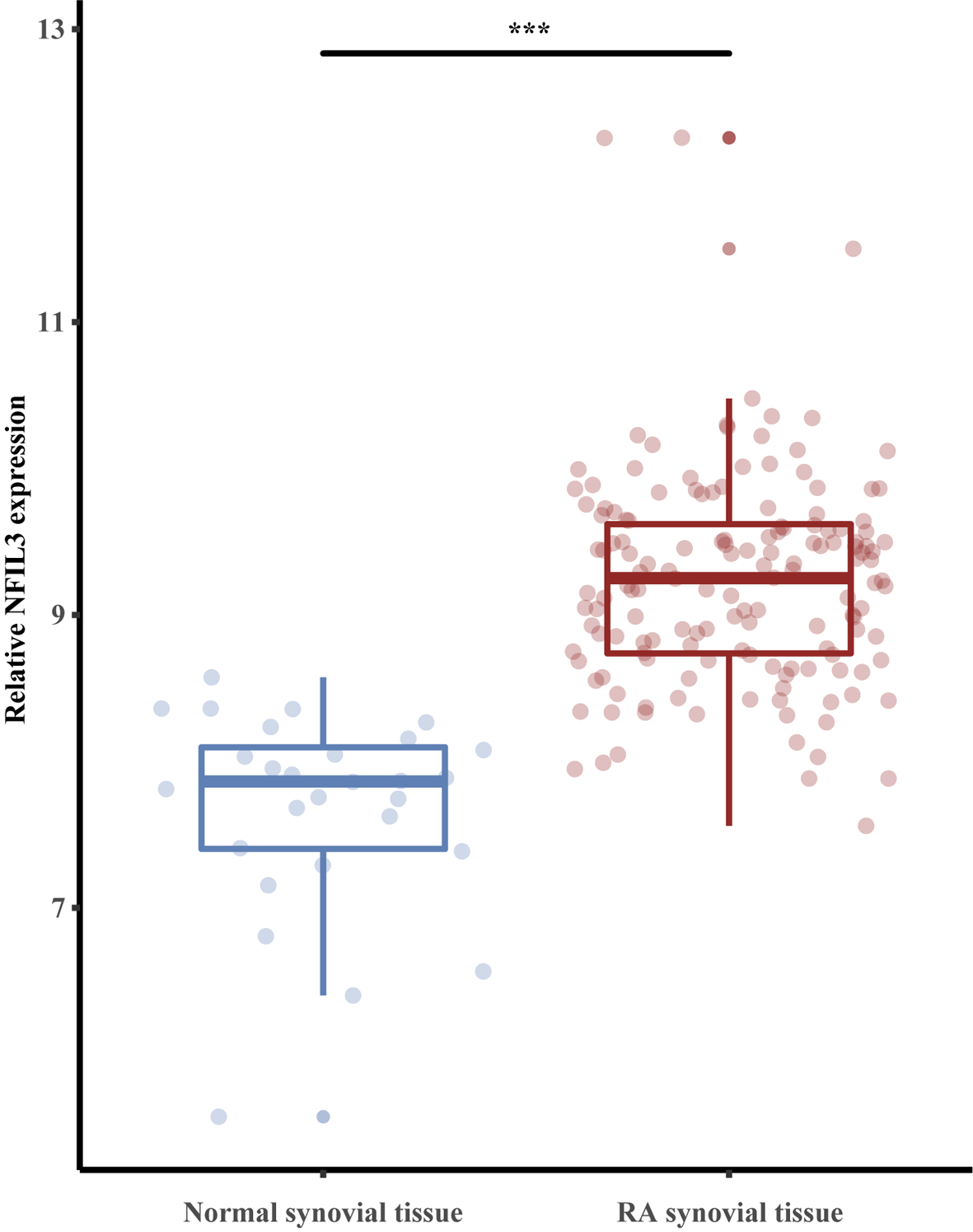

Supplement: Supplementary Figure 1 — Up-regulation of NFIL3 expression in RA synovial tissues compared with normal tissues from the GSE89408 dataset. [file Image_1.tif]

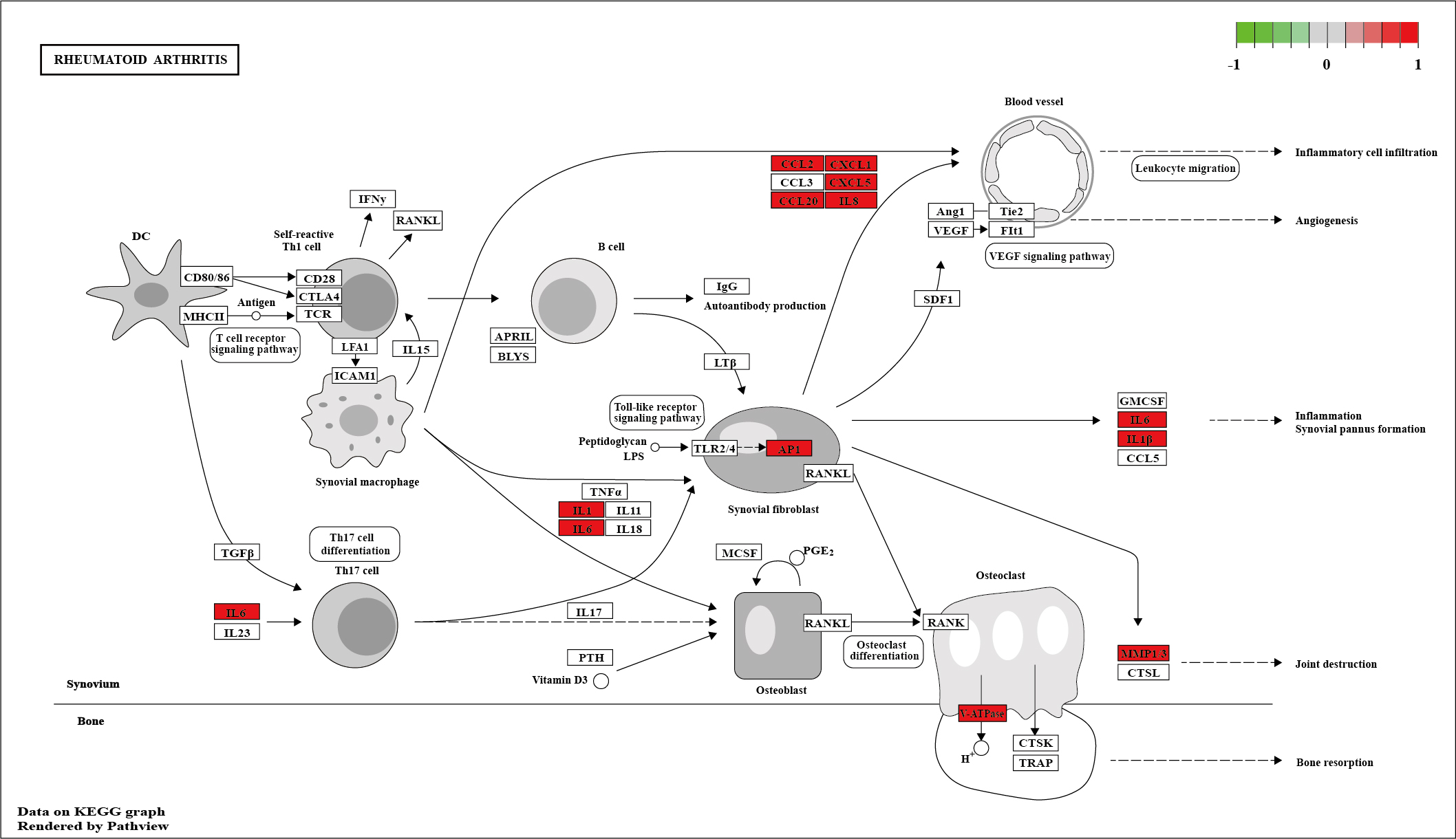

Supplement: Supplementary Figure 2 — Rheumatoid arthritis pathway between NFIL3-high and NFIL3-low groups. The expression changes of DEGs related to the hsa05323 rheumatoid arthritis pathway were visualized by online database Pathview. Red indicated increased expression. [file Image_2.jpeg]

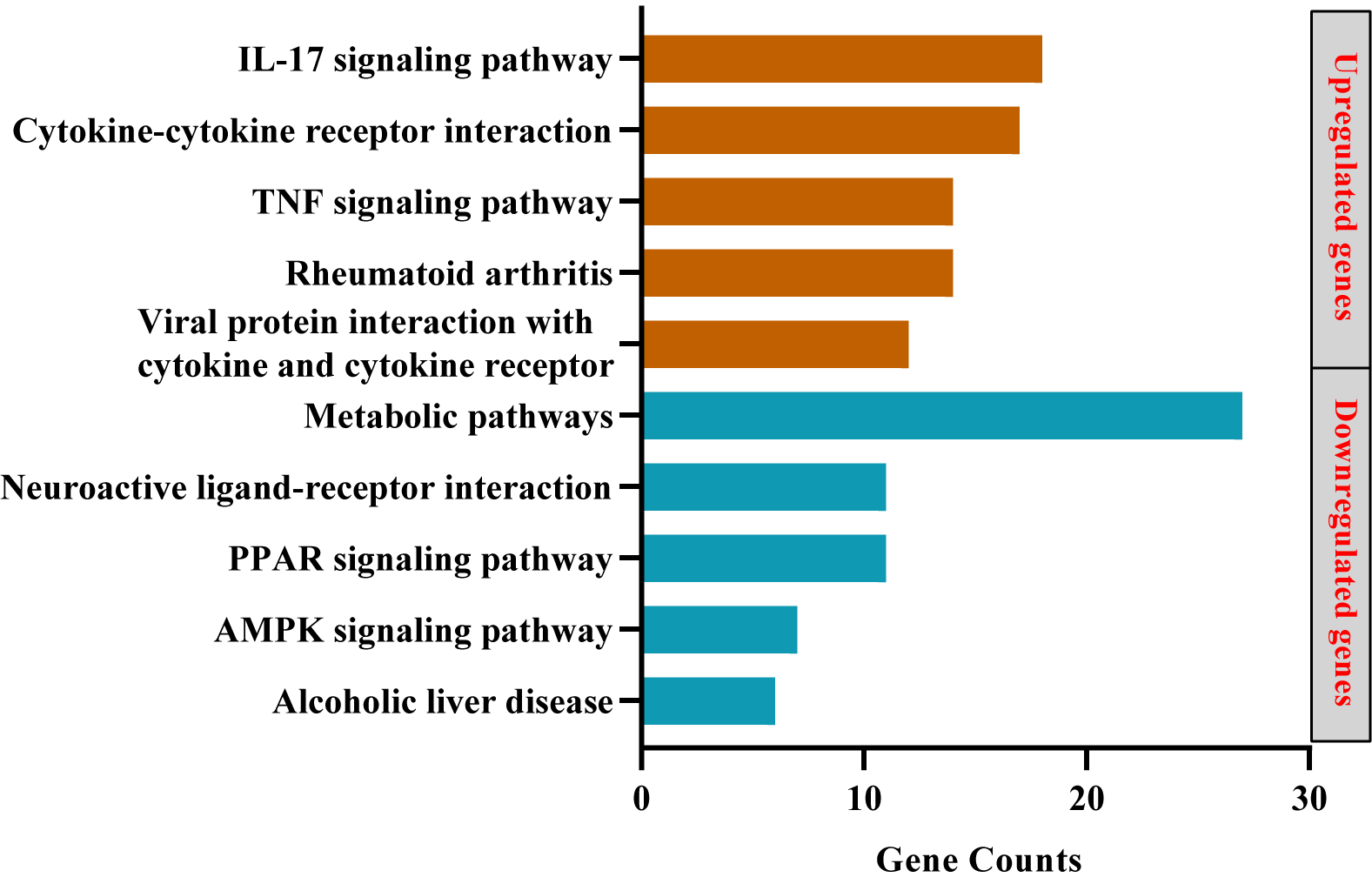

Supplement: Supplementary Figure 3 — KEGG analysis of the upregulated and downregulated DEGs. Up-regulated DEGs were mainly enriched in IL-17 signaling pathway and cytokine-cytokine receptor interaction pathways. Down-regulated DEGs were mainly enriched in metabolic pathways. [file Image_3.tif]

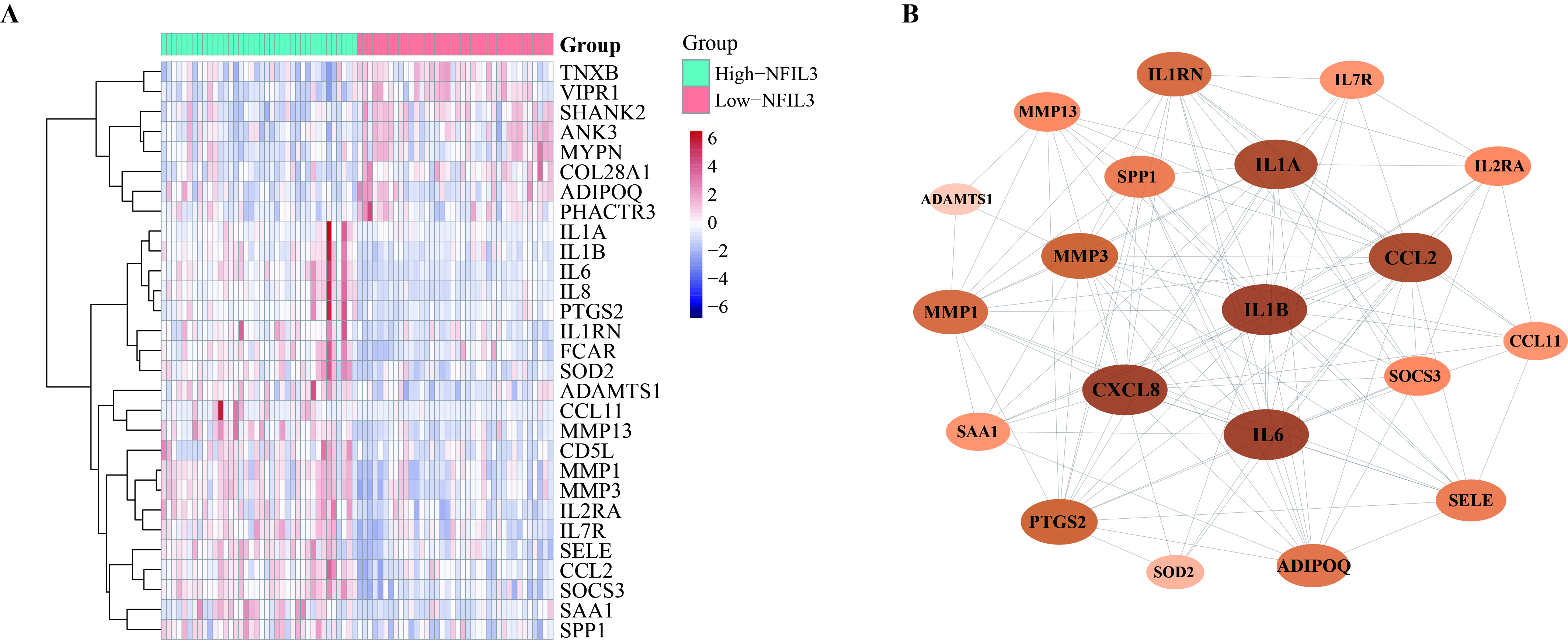

Supplement: Supplementary Figure 4 — The heatmap and PPI network of 29 RA- and NFIL3-related genes. There are 29 overlapping genes between the NFIL3 co-expression genes and the RA-related genes from the RADB database. (A) A heatmap was applied to visualized the expression differences of 29 RA-related genes in the NFIL3-high and NFIL3-low groups. Blue indicates down-regulation; red indicates up-regulation. (B) The PPI network contains 20 nodes and 117 edges. The expression of all the nodes were upregulated. [file Image_4.jpeg]
